# Varied presentations of complicated rhinosinusitis in COVID era: a rational approach to management

**DOI:** 10.1186/s43163-022-00374-z

**Published:** 2023-01-09

**Authors:** Shivali Thakur, Uma Patnaik, Pavitra Saxena, Manvir Singh Tevatia, Gunjan Dwivedi, Abha Kumari, Nusumu Purnachandra Rao, Amit Sood

**Affiliations:** 1 Department of ENT, Command Hospital Pune, Pune, India; 2 Department of ENT, Armed Forces Medical College, Pune, India; 3 Command Hospital Pune, Pune, India

**Keywords:** Rhinosinusitis, Mucormycosis, Histopathological examination, Intraorbital complications, Amphotericin B, Uncontrolled diabetes

## Abstract

**Aim:**

To study the various presentations and manifestations of complicated rhinosinusitis in COVID era- ranging from bacterial rhino sinusitis to invasive fungal rhino sinusitis.

**Methods:**

Design-A retrospective observational study was carried out from March 2020 to May 2021. Setting-Tertiary care hospital subjects—all COVID-positive patients who had paranasal sinus involvement. Methods-Patients were evaluated based on their symptomatology profile. Fungal stains and culture were carried out for all. They underwent Magnetic resonance Imaging and Computed Tomography scan on case-to-case basis, apart from routine nasal endoscopy. All were managed both medically and surgically depending upon their diagnosis. The natural course including outcomes, was studied, documented and analyzed.

**Results:**

Out of 496 patients presenting with sinonasal disease, 126 were COVID-positive, 16 patients had complicated rhino sinusitis, of which 4 patients had complicated rhinosinusitis with intraorbital, intracranial or combined complications. All patients were managed successfully with combined medical and surgical approach. Twelve patients had invasive mucormycosis with overall mortality rate of 37%.

**Conclusion:**

Complicated sinusitis was encountered in COVID-positive patients either when they were being actively treated for COVID-19 or as part of post-COVID sequalae. Though rhino-orbito-cerebral mucormycosis constituted the major disease burden in such patients but the possibility of bacterial rhino sinusitis with or without complications must also be kept in mind while evaluating such patients. We must remember every complicated rhinosinusitis in COVID-positive patient may not be mucor and manage appropriately.

## Background

The COVID-19 infection caused by the novel severe acute respiratory syndrome coronavirus 2 (SARS-CoV-2) has been the latest pandemic; the whole world is going through. The first COVID case was detected and documented from the province of Hubei, Wuhan, China on 01 December 2019 [1]. In India, the first case was reported on 27 Jan 2020 from Kerala [2]. However, in our tertiary care hospital, the first case was admitted on 29 July 2020. During the current pandemic of COVID-19, a myriad of manifestations and complications have emerged and are being reported on. The involvement of nasal and paranasal sinuses is emerging in a big manner in overall COVID-19 symptomatology with anosmia without associated nasal obstruction being reported as a very important predictor of COVID-19+ patients. The sinonasal cavity might be considered as a major site of infection by the deadly SARS-CoV-2, owing to higher levels of expression of the concerned susceptibility genes. This in turn is modulated by the various environmental and host factors. Viral load and shedding appear to be the highest from nose and paranasal sinuses, hence forming a major chunk in clinical presentations and transmission in cover positive patients [3].

The importance of classical rhinosinusitis and its potential to cause various infraorbital and intracranial complications needs to be re-emphasized in this present COVID-19 era [4–6]. Chronic rhinosinusitis can also exacerbate the pre-existing comorbid pulmonary conditions, including COVID pneumonia. And in this COVID-19 era, the management of such complicated rhino sinusitis cases has become far more challenging.

In last one year, however, both our experience and constant threat of the SARS-COV2 virus has led to certain changes in our conventional practice of treating rhino sinusitis and its complications, resulting in change of our own institutional protocol for the same. Wearing PPEs, use of negative pressure rooms for performing any aerosol generating procedure, encouraging use of nasal pledges rather than sprays for instilling topical nasal drugs, restricting nasal endoscopies, favoring imaging studies as compared to diagnostic nasal endoscopy in doubtful cases, wearing mask and performing endoscopy through a small hole are few of the improvisations being followed for treating such cases nowadays. Needless to say, this Corona pandemic has resulted in a great paradigm shift in managing sinonasal infections [7–9].

There are many studies being published regarding rising Mucormycosis cases in COVID and post-COVID patients. But we wanted to emphasize on the fact that apart from Mucor infection, other sinonasal disease spectrum can also present in this COVID era. Hereby, we present a retrospective analysis of our experience of 16 cases of nose and paranasal disease with either COVID-positive status or its sequelae who were managed at our institution. In this study, we have tried to discuss and analyze about the entire spectrum of cases of sinonasal infections ranging from rhinosinusitis with intraorbital or intracranial complications to invasive rhinocerebrorbital mucormycosis in COVID-positive patients.

## Methods

A retrospective observational study was carried out at a tertiary care hospital of armed forces from March 2020 to May 2021. During this period, a total of 496 cases of sinonasal infections were encountered and treated. Out of 496 cases, 126 cases were COVID-positive. Out of these 126 cases, 16 cases were admitted, diagnosed, and managed as cases of complicated rhinosinusitis including invasive fungal rhinosinusitis. Thus, a total of 16 cases consisting of 1 pediatric case and 15 adults’ patients of sinonasal disease ranging from rhinosinusitis with complications to invasive fungal rhinosinusitis (including mucormycosis) were finally included in the study. This study was compliant with the Helsinki declaration, hence exempted from obtaining ethics committee approval being a retrospective study. Written informed consent was taken from all the patients.

### Case definition and symptomatology

Patients with disease confined to nose and the paranasal sinuses were defined as having rhinosinusitis; those with disease in the paranasal sinuses and infiltrating the orbit were defined as having sino-orbital infection; those with disease in the paranasal sinuses and the brain were categorized as having rhino cerebral infection, with cerebral involvement defined as tissue invasion demonstrated radiologically with intracranial evidence of disease by either Computed tomography (CT) or Magnetic resonance imaging (MRI), or severe neurological impairment.

All patients who presented after March 2020 with one or more of following symptoms: headache, facial swelling/cellulitis, peri-orbital edema, sudden loss of vision, and palatal bone/mucosa necrosis were included in the study. Documentation of the primary underlying condition or of immunosuppression was documented for each reported case, unless the patient was described as having no underlying condition.

#### Inclusion criterion

Patients presenting with symptoms suggestive of sinonasal disease (as mentioned above)

#### Exclusion criterion

COVID-negative patients

### Investigations and radiology

All patients underwent reverse transcriptase polymerase Chain reaction (RT-PCR) test for COVID-19 upon admission as a standard protocol. All positive patients were treated in dedicated acute COVID wards. Only COVID-positive cases were included in this study. History of prolonged steroid use and Oxygen support (mode and rate) was taken. Routine hematological investigations, nasal swabs for bacterial and fungal staining, and cultures were carried out and any other special investigations (based on specific patient needs) were also ordered. Detailed computed tomography (CT) or Magnetic resonance imaging (MRI), examination, and diagnostic nasal endoscopy was carried out for all patients and findings documented. All patients underwent MRI to look for intraorbital and intracranial complications and CT scan for seeing bony involvement and for surgical planning

### Treatment

**Fig. 1:**
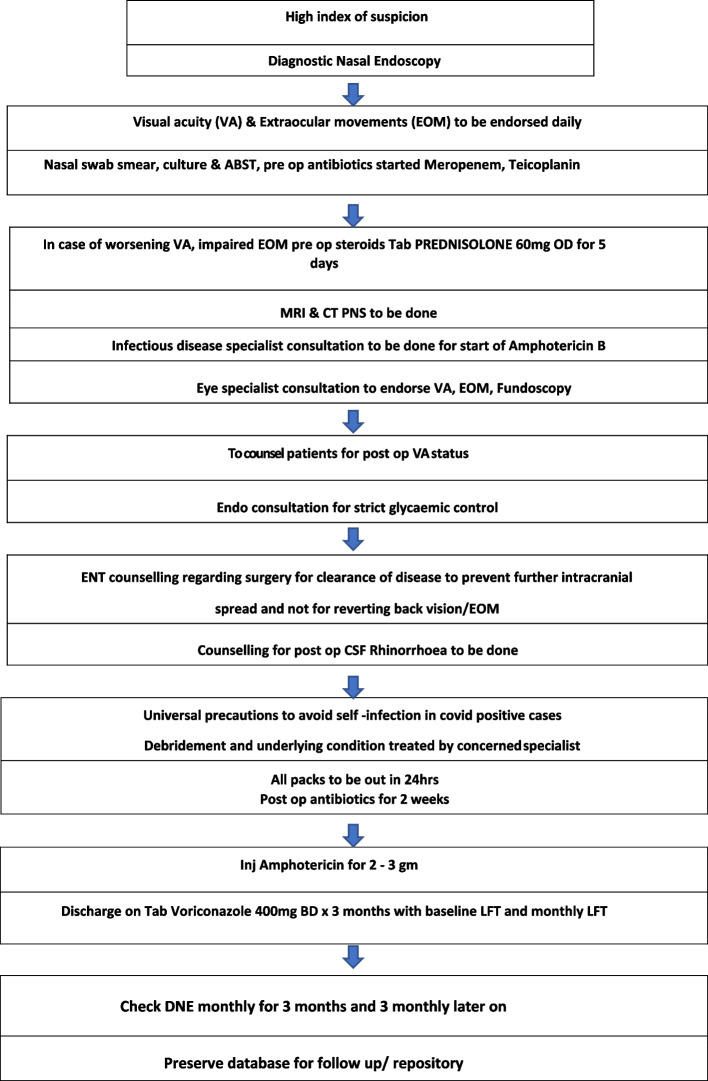
Mucormycosis management protocol

All patients were managed as per our institutional protocol by multispeciality team (ref Fig. 1). Every patient was jointly assessed by ENT specialist, infectious disease specialist, ophthalmologist, neurologist, intensivist, and microbiologist. A session of group counselling was conducted for all patients and their next of kin. The medical therapy including the antifungal therapy (e.g., Liposomal Amphotericin B) was started after consulting infectious disease specialist. The surgical intervention varied from functional endoscopic sinus surgery (FESS) to maxillectomy (partial/total) and orbital exenteration in selected cases of invasive fungal rhinosinusitis (IFRS). Subsequent management was dependent upon the progress of the patient on follow up.

### Statistical analysis

The main categorical variables studied included age, sex, known comorbidities, diabetes (type and presence of ketoacidosis), infecting organism, diagnostic method used, infection site (focal or disseminated disease), medical treatment, surgical management, and oxygen therapy for COVID and outcome (refer to Table 1).

**Table 1:** Case details

Case series type	Age/gender	Comorbidities	Treatment (medical/surgery or both)	Outcome
Case series type 1	15/M	Mild COVID infection	Both	Complete recovery
Case series type 2	10/M	Post-COVID sequelae	Both	Complete recovery
Case series type 3	38/M	Poorly controlled diabetes mellitus Post-COVID sequelae	Both	Recovery with persistence of III, IV, V1, V2, VI, VII cranial nerve palsies
	55/F	Poorly controlled diabetes mellitus Post-COVID sequelae	Both	Complete recovery
Case series type 4	63/F	Poorly controlled diabetes mellitus Post-COVID sequelae	Both	Complete recovery
	44/M	Poorly controlled diabetes mellitus Post-COVID sequelae Chronic liver disease	Both	Succumbed to his illness
	35/M	Poorly controlled diabetes mellitus Severe COVID pneumonia DKA	Both	Succumbed to his illness
	40/M	Poorly controlled diabetes mellitus Moderate COVID pneumonia	Both	Succumbed to his illness
	54/M	Poorly controlled diabetes mellitus Moderate COVID pneumonia	Both	Complete recovery with resolution of symptoms
	62/F	Poorly controlled diabetes mellitus Post-COVID pneumonia HTN	Both	Complete recovery
	58/F	Poorly controlled diabetes mellitus Severe COVID pneumonia DKA HTN	Medical	Died before surgical intervention
	66/M	Poorly controlled diabetes mellitus Post-COVID pneumonia	Both	Complete recovery
	58/M	Poorly controlled diabetes mellitus Post-COVID pneumonia	Both	Recovery with persistence of complete ophthalmoplegia
	55/M	Moderate COVID pneumonia	Medically	Died before surgical intervention
	48/M	Moderate COVID pneumonia	Both	Recovering
	42/F	Moderate COVID pneumonia	Both	Succumbed to her illness

All the findings were documented and recorded. The data from all patients was compiled, analyzed and inferences /results were drawn.

### Ethical consideration

This study was compliant with the Helsinki declaration, hence exempted from obtaining ethics committee approval being a retrospective study. Written informed consent was taken from all the patients.

## Results

Out of 496 patients presenting with sinonasal disease, 126 were COVID-positive, 16 patients had complicated rhino sinusitis, of which 4 patients had complicated rhinosinusitis with intraorbital, intracranial, or combined complications. All patients were managed successfully with combined medical and surgical approach. Twelve patients had invasive mucormycosis with overall mortality rate of 37%.

### Demographic profile

Out of our total 16 cases, 5 (31.25%) were females, 11 (68.75%) were males. Only 1 (6.25%) patient was from pediatric population rest all were adults. Of all the 16 cases, 5 (31.25%) had single comorbid condition, 6 (37.5%) had more than one underlying comorbidity, and 5 (31.25%) had no comorbidity.

### COVID status

All the patients included in our study were COVID affected. Eight (50%) patients had recovered from COVID pneumonia whereas 8 (50%) had ongoing COVID infection. Out of 8 patients suffering from recent COVID infection, 1 (12.5%) had mild infection, 5 (62.5%) had moderate COVID pneumonia, 2 (25%) had severe COVID pneumonia. Out of 8 recovered patients, 1 (12.5%) had mild pneumonia, 5 (62.5%) had moderate COVID infection, 2 (25%) had severe COVID pneumonia. Out of all the 16 patients, 12 (75%) were given supplemental oxygen at rates depending upon their condition and response. Twelve (75%) patients out of all the 16 were given steroids for the management of COVID.

### Case details

#### Case series prototype 1—complicated rhinosinusitis with intracranial extension without intraorbital involvement

**Fig. 2:**
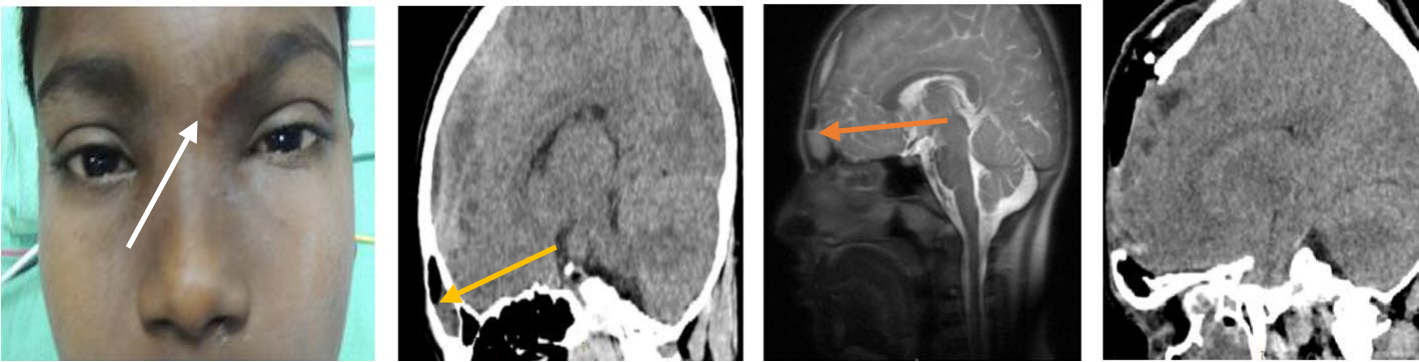
**a**–**d** Representative images of case series 1. **a** Clinical picture of the patient with a 2 × 1 cm tender swelling above (L) eyebrow. **c** (R) frontal sinus collection extending up to® frontal lobe with (R) frontal lobe abscess on CT scan. **d** Extradural collection in frontal region in midline with (R) frontal lobe abscess on MRI. **e** Post-surgery CT scan after wide bifrontal decompressive craniectomy

One patient complicated rhinosinusitis with intracranial extension without intraorbital involvement in a COVID-positive male patient aged 17 years without any known comorbidities. His presenting complaints were fever, (L) nasal obstruction, (L) rhinorrhoea X 6 days following trauma to head, which was followed by 2 episodes of vomiting, headache and acute onset (R) hemiparesis. On DNE, there was mucopus in the left middle meatus. His KOH mount was negative for fungal elements, CT scan showed extra-axial collection in frontal region with hyperdense opacity in (L) frontal, maxillary, and ethmoidal sinus. His MRI showed extra dural collection in frontal region in midline with (L) maxillary sinusitis which was suggestive of abscess. He was managed both medically and surgically. He underwent endoscopic sinus surgery along with wide bifrontal decompressive craniectomy with evacuation of pus from frontal lobe (refer to Table 1 and Fig. 2). Post-operative histopathological examination (HPE) report showed non-specific inflammation. He responded well to surgery with complete resolution of signs and symptoms in next 1 year.

#### Case series prototype 2—complicated rhinosinusitis with intraorbital extension without intracranial involvement

**Fig. 3:**
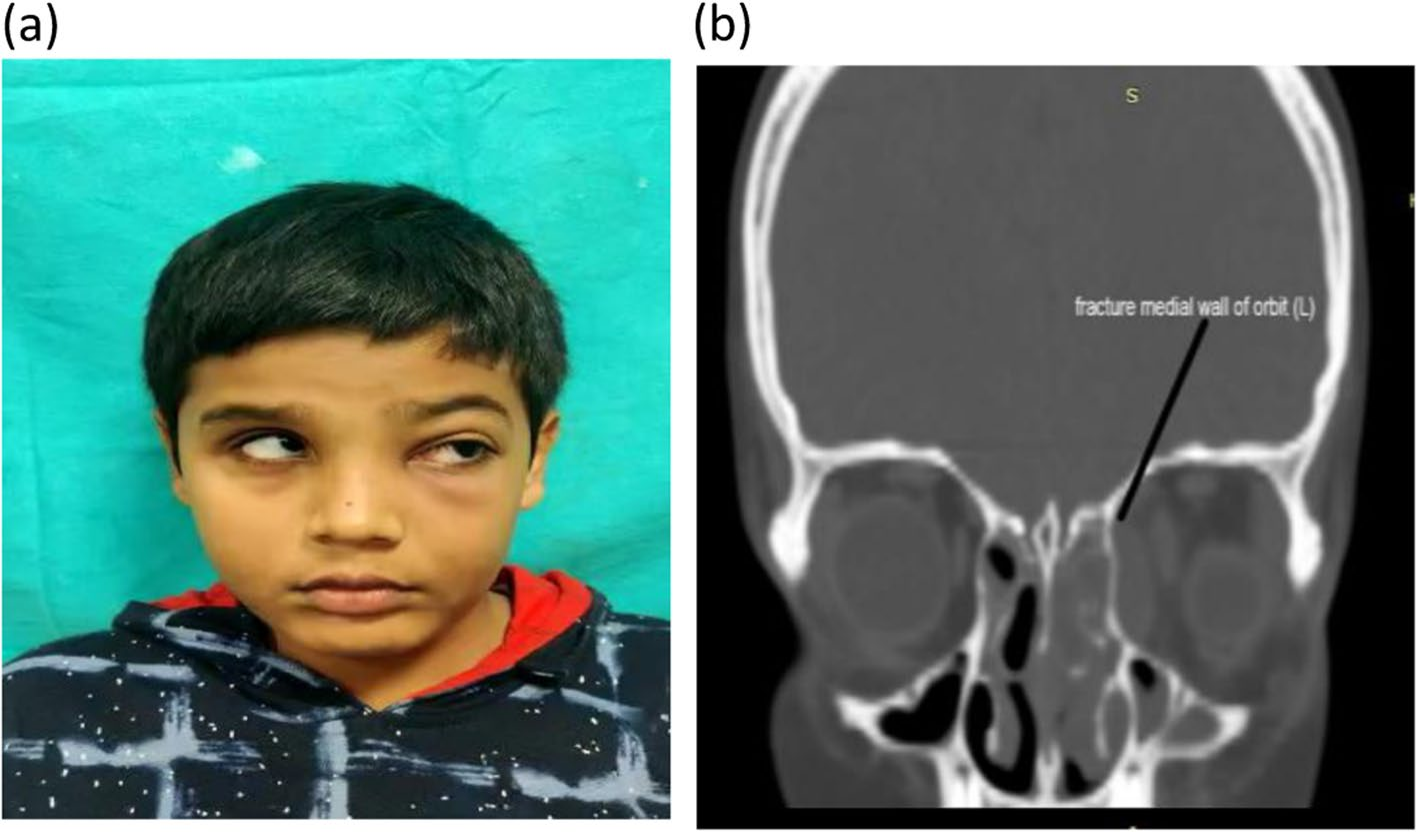
**a**, **b** Representative images of case series 2. **a** (L) periorbital swelling, mild erythema, Proptosis (L) eye, restricted superior gaze in a case of complicated rhinosinusitis with subperiosteal abscess. **b** CT PNS image showing fracture of medial wall of (L) orbit, subperiosteal hematoma along medial wall of (L) orbit with proptosis, indentation of (L) medial rectus. Hemosinus in (L) ethmoidal air cells and mucosal thickening involving all paranasal sinuses

One patient had complicated rhinosinusitis with intraorbital extension. The patient was a 10-year-old male with no comorbidities. He had a history of post-COVID pneumonia. He presented to us with a history of fall from bed and trauma to (L) orbital region and nose which was proceeded by one episode of vomiting and fever after one day and B/L rhinorrhoea and swelling over (L) eye after 2 days. On diagnostic nasal endoscopy (DNE), there was a swelling (L) lateral wall touching septum and B/L congested mucosa. On eye examination, there was (L) periorbital swelling, mild erythema, proptosis (L) eye, superior gaze restricted, tenderness (L) supraorbital region, supra and infra orbital ridges well palpated. His CT scan showed subperiosteal hematoma along the medial wall of (L) orbit with proptosis, indentation of (L) medial rectus, hem sinus (L) ethmoidal air cells, and mucosal thickening involving all paranasal sinuses (refer Table 1 and Fig. 3). He was managed as a case of acute bacterial rhinosinusitis with subperiosteal swelling (L) eye medically and surgically by extended endoscopic sinus surgery (EESS) and had complete recovery.

#### Case series prototype 3—complicated rhinosinusitis with intraorbital and intracranial extension

**Fig. 4:**
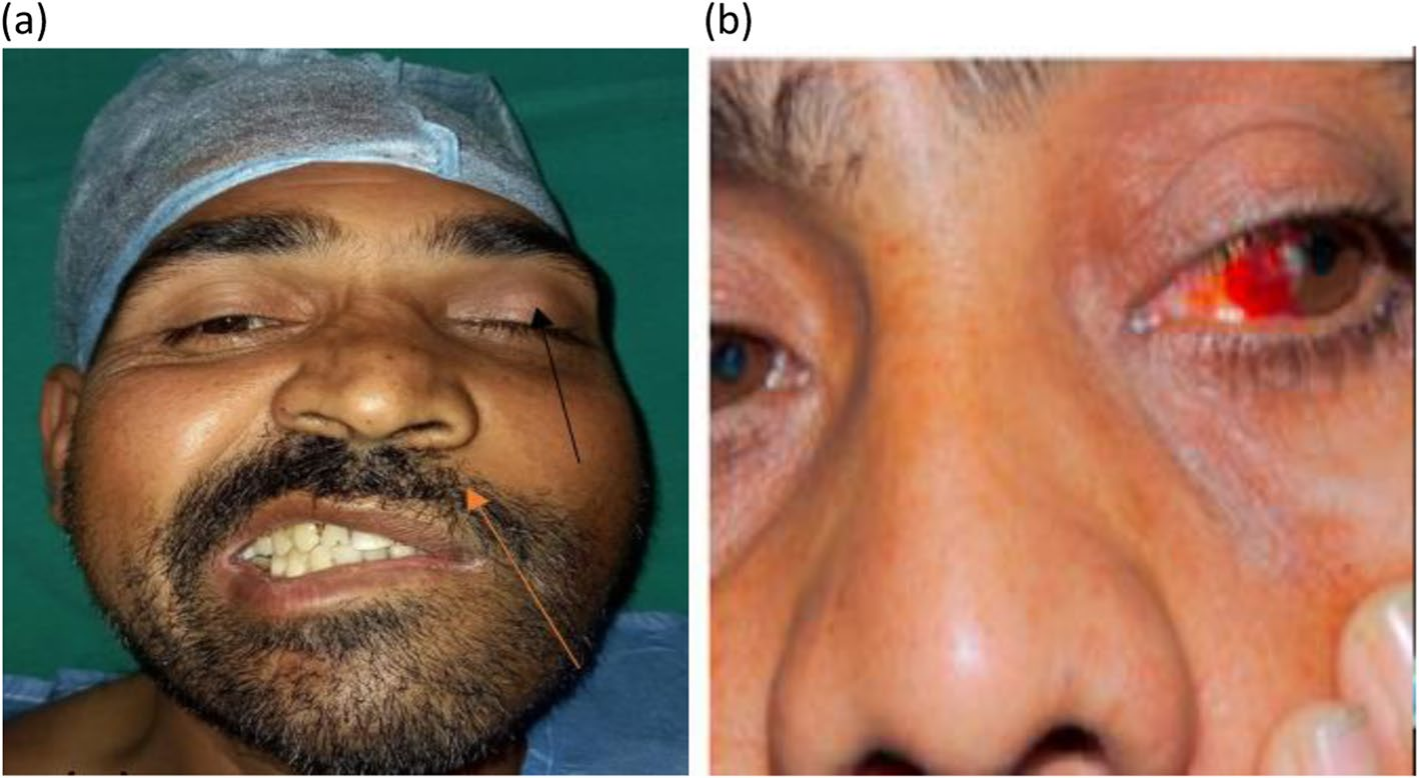
**a**, **b** Images of illustrative case from case series 3. **a** (L) LMN type House Brackmann Gd V facial palsy with complete ophthalmoplegia (L) and ptosis (L). **b** Red eye (L) with diminution of vision in a case of rhinosinusitis with intracranial and intraorbital complications

**Fig. 5:**
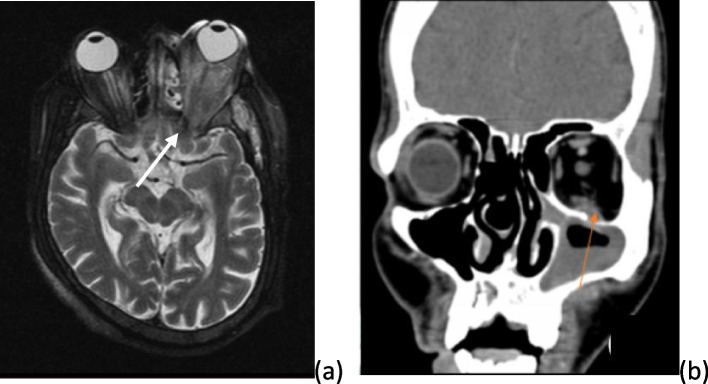
**a**, **b** Radiographic pictures of case series 3. **a** Thickened superior ophthalmic vein with possible cavernous sinus thrombosis on MRI. **b** CT image showing opacities in (L) maxillary sinus extending into (L) orbit through a breach in the orbital flo

There were 2 patients fitting into the diagnosis of complicated rhinosinusitis with intraorbital and intracranial extension. 1 patient was a 38-year-old male and other was female patient aged 55 years. Both had post-COVID sequelae. Both were known diabetics with poor glycemic control despite of aggressive medical management by endocrinologist. The female patient had cavernous sinus thrombosis secondary to rhinosinusitis and the male patient had rhinosinusitis with complications in the form of multiple cranial nerve palsies and multiple abscess Infratemporal Fossa (ITF) and pterygoid fossa. Both the cases showed opacities in the paranasal sinuses with cavernous sinus involvement (refer to Figs. 4 and 5 for illustrative case) (refer to Table 1). Both were managed medically followed by surgical intervention in the form of endoscopic sinus surgery. Both had an excellent result.

#### Case series prototype 4—invasive fungal rhinosinusitis (IFRS)

**Fig. 6:**
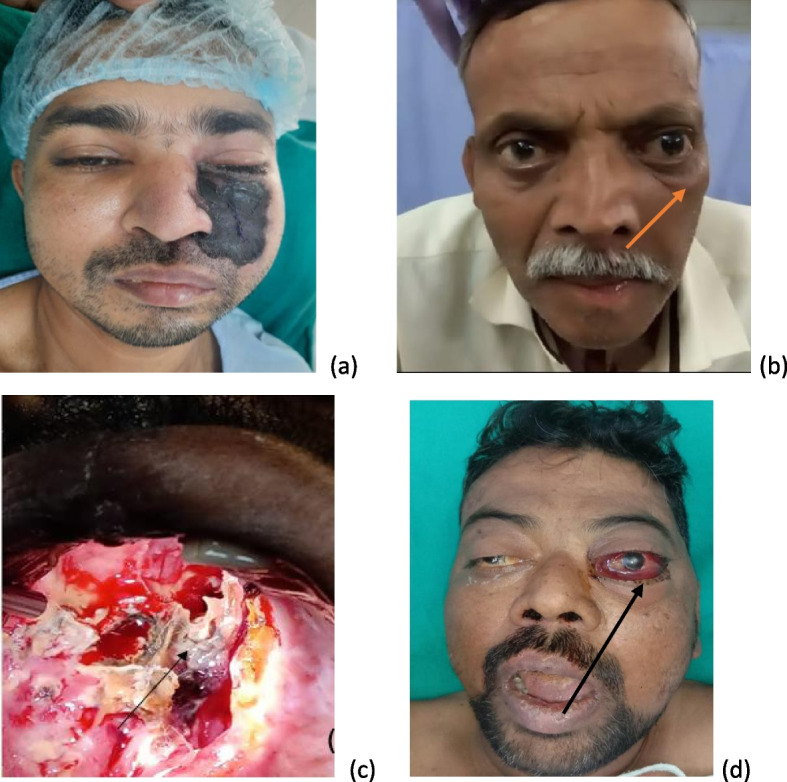
**a**–**d** Images of representative cases from case series prototype 4. **a** Eschar over (L) cheek in a case of cutaneous mucormycosis. **b** Incomplete ophthalmoplegia with restricted eye movements in medial and superomedial quadrants in case of invasive fungal rhinosinusitis with (L) intra orbital extension. **c** An intraoperative picture of a patient undergoing maxillectomy for palatal involvement in rhino-orbito-cerebral mucormycosis. **d** Severe chemosis, proptosis with complete ophthalmoplegia (L) eye in a case of rhino orbital cerebral mucormycosis

**Fig. 7:**
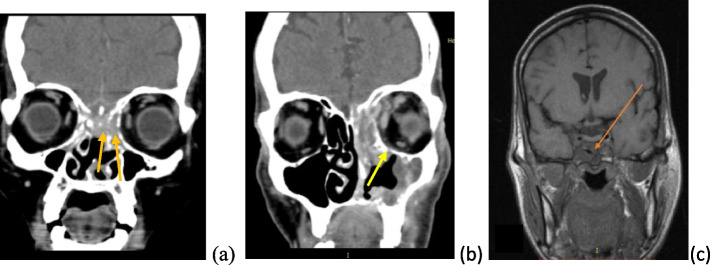
**a**–**c** Radiological investigations of prototype cases of case series 4. **a** CT PNS shows hypodense opacities in bilateral ethmoids with erosion of cribriform plate bilaterally. **b** CT PNS of a patient with rhino orbital mucormycosis showing (L) intra orbital abscess with defect in cribriform plate. **c** MRI (T1 weighted) image of a patient with rhino orbital cerebral mucormycosis with cavernous sinus thrombosis (R)

In a series of 12 cases (4 females, 8 males) with mucormycosis (confirmed by microbiological and HPE in all clinically suspected cases), 9 patients had uncontrolled diabetes. Out of all the 9 diabetics, 3 had ketoacidosis. Two patients had chronic kidney disease; 1 patient was a known case of alcohol related chronic liver disease whereas 3 were hypertensive. Out of total 12 patients, 4 patients had more than one comorbid condition. A total of 6 patients had history of prior COVID infection and 6 had ongoing COVID pneumonia. Out of 6 patients who presented as post-COVID sequelae, 3 had severe disease, rest of 3 had moderate pneumonia. Amongst the 6 patients who had an ongoing COVID infection, 4 had moderate illness whereas 2 had severe disease. All the moderate and severe COVID-infected patients were kept on supplemental oxygen support depending upon their condition and response to therapy. All the COVID-infected patients were given steroids. Out of these 12 confirmed mucormycosis patients, 6 underwent endoscopic sinus surgery with debridement of necrotic tissue, 1 had undergone ESS with debridement of necrotic tissue along with orbital exenteration and maxillectomy, 1 underwent ESS with debridement of necrotic tissue along with orbital decompression, 2 expired before commencement of any surgical intervention within 24 h of their presentation, rest 2 died while being worked up surgery. Six patients having very high index of suspicion were started on injection L-Amp B in the first instance empirically after consulting infectious disease specialist, rest 6 patients were given L-Amp B after microbiological/ histopathological confirmation. Five out of all 12 mucormycosis patients had favorable outcomes in terms of resolution of clinical symptoms, negative biopsy report for fungal hyphae, 5 patients succumbed to their illness, 2 patients are still recovering. The overall fatality in mucormycosis cases at our center in the last 1 year was 41.6%.

(For detailed description of patient’s presenting complaints and clinical examination findings refer Table 1 and ref Figs. 6 and 7 for representative cases)

### Treatment and outcome

Of the 16 patients included in the study, 14 (87.5%) patients were treated both medically and surgically, 2 (12.5%) died before any surgical intervention. The overall complete recovery was shown by 7 (43.75%) patients, 2 (12.5%) patients recovered with persistence of some neurological deficit, 6 (37.5%) patients died, 1 (6.25%) patient is recovering. The overall fatality in our case series was 37.25%.

## Discussion

COVID-19 is associated with a significant incidence of secondary infections, both bacterial and fungal probably due to immune dysregulation. Additionally, the widespread use of steroids/monoclonal antibodies/broad-spectrum antibiotics as part of the armamentarium against COVID-19 may lead to the development/exacerbation of preexisting sinonasal infections, therefore, it might be suggested that SARS-CoV-2 infection by itself can induce an immunosuppressive state that exposes the patient to the risk of developing opportunistic infections, such as mucormycosis.

### Pathophysiology

Besides, the diffuse alveolar damage with severe inflammatory exudation, COVID-19 patients always have immunosuppression with a decrease in CD4 + T and CD8 + T cells. Critically ill patients, who require mechanical ventilation, or longer duration of hospital stays are more likely to develop fungal sinonasal co-infections. The main fungal pathogens documented in literature are *Aspergillus* and *Candida*; however, COVID-19 patients with uncontrolled diabetes mellitus (persistent acidotic state), glucocorticosteroids (GC) use in moderate-severe COVID infections, requirement of prolonged humidified oxygen therapy, are more likely to develop mucormycosis, which can be life threatening to the patient. Various other immunocompromised states in an individual namely diabetic ketoacidosis, hematological malignancies, neutropenia, solid organ malignancies, high dose corticosteroids, post-organ transplantation, deferoxamine therapy, trauma, and burns also act as predisposing factors for mucormycosis. A study of 929 cases of zygomycotic showed prevalence and mortality of 36% and 44% resp. in patients with underlying diabetes [10, 11]. The immunocompromising effects of corticosteroids with microangiopathy in diabetic patients and possible peripheral microthrombi in COVID-19 provide the ideal host for invasive fungal rhinosinusitis, which due to its inherent angioinvasive nature leads to its increased incidence in such patients. Tissue necrosis, often a late sign, is a hallmark of mucormycosis, resulting from angioinvasion and vascular thrombosis.

Hyperglycemia especially along with acidosis is an ideal environment for fungal growth which is aided by weakened immune system secondary to altered chemotaxis and ineffective phagocytosis of PMNs and macrophages resp. due to impaired glutathione pathway [12, 13]. For Mucorales, iron appears to be a growth factor. Normally, transferrin, a serum iron binding protein clubs with iron and deprives the fungus of the same thus inhibiting their growth. In such situations microbes secretes siderophores which binds with the serum iron and in turn is utilized by the growing fungus. The excessive glycosylation of transferrin and ferritin protein in cases of uncontrolled diabetes leads to its decreased affinity for iron and thus its free availability to the growing fungus [14]. Deferoxamine, an iron and aluminum chelator in humans used in cases of iron overload in renal patients on dialysis also acts as a false siderophore for Rhizopus thus enabling their iron uptake and augmenting their hyphal growth [15, 16]. Patients with hematological malignancies, solid organ, or hematopoietic stem cell transplant (HSCT) recipients, which are continuously under the effects of immunosuppressants also lead to increase in the incidence of mucormycosis infection.

### Comparison with other studies

Very few reports in literature have been documented so far, wherein complicated sinusitis has occurred in patients having concomitant COVID-19 infection [17]. One of the main pathophysiological mechanisms described is the occurrence of a generalized pro-thrombotic state resulting into microvascular and macrovascular thromboembolism. Han et al. wrote that certain coagulation parameters like raised D-dimers, fibrin degradation products, and fibrinogen correlate with severity of COVID-19 infection [18]. The pathophysiology of thrombophilia in SARS-CoV-2 infection is still debatable; however, some mechanisms have been proposed, which includes a pro-coagulable cytokine storm, role of viral affinity for angiotensin converting enzyme 2 (ACE2) receptors situated in vascular endothelium and also raised antiphospholipid antibodies. Reports of complications like pulmonary embolisms, deep vein thrombosis, cerebral infarction, and cerebral venous sinus thrombosis associated with ongoing COVID-19 infection are increasing in recent literature [19]. This may explain the intracranial complications including a case of cavernous sinus thrombosis in two patients of our case series.

In our review of literature, we came across various case reports but very few case series reporting complicated rhino sinusitis in COVID-positive patients. Despite the tropism of COVID-19 to both upper and lower respiratory mucosa, a very limited number of reports have studied its association with the development of complicated sinusitis. Turban et al. reported two cases of COVID-19-associated complicated sinusitis with orbital cellulitis [20].

White et al. reported an incidence of 26.7% for invasive fungal infections in his case series of 135 adults having COVID-19 infection [21]. Song et al. described association between COVID-19 and invasive fungal sinusitis in April 2020, and came to conclusion that a large number of patients either affected by or recovered from COVID-19 are at greater risk of developing invasive fungal diseases, and also devised a management algorithm for such cases [14, 22].

### Pediatric rhinosinusitis

Disease spectrum of COVID-19 in pediatric population is a gray zone. Xu et al. found that patients in pediatric age group were 2.7 times less likely to contract the illness as compared to adults. Although data has suggested less severe disease form in the pediatric population, but still pediatric mortalities have been documented in the USA [23]. Conor H et al. have described two different approaches for dealing with complicated pediatric rhinosinusitis in COVID-positive patients in their case series [24]. Though we saw and successfully managed only one case of complicated CRS in a 10-year-old boy, but we hope that even a small contribution may result in a better understanding of disease progression in children.

### Invasive fungal rhinosinusitis

Mucormycosis is a highly aggressive and relentlessly progressive invasive fungal infection with high mortality rates and its incidence has tremendously increased in patients who are suffering from COVID-19 or just recovered from it. In diabetic patients who are being treated for moderate-severe COVID Disease, Mucor infection has become very rampant owing to acidotic state providing an excellent media for mucor growth. Majority of cases have no prior history suggestive of rhino sinusitis. The intraorbital invasion by Mucor often leads to irreversible loss of vision as a part of post-COVID sequelae, making it famous as black fungus. The dilemma lies in distinguishing opportunistic invasive Mucor from other commensal fungus in nose and paranasal sinuses, thus highlighting a very important role of molecular biology, and other pathological and microbiological tests. Rhizopus, Mucor, Rhizomucor, Absidia, Saksenaea, Apophysomyces, and Cunninghamella are the commonly recovered genera with Rhizopus *oryzae* as the most frequent pathogen associated with mucormycosis and is being accounted for approximately 60% of all the mucormycosis infections and a contributor of approximately 90% of rhino cerebral cases [25, 26].

### Management in a nutshell

These sinonasal infections manifest themselves more commonly in immunocompromised states, thus requiring a multispeciality involvement and treatment of comorbid conditions apart from treating the primary sinonasal pathology. Since appearance of tissue at endoscopy as well the radiological findings lag behind the clinical progression of the disease, a prompt biopsy should be taken keeping a high index of suspicion in order to promptly diagnose and instigate the treatment at the earliest. The principle of management of any sinonasal disease depends upon four factors namely (a) rapidity of diagnosis (b) reversal of underlying predisposing factors, if any (c) surgical debridement (d) appropriate medical therapy including antibiotics and antifungals [27–32]. Early diagnosis is an important factor as a small lesion can be effectively treated surgically prior to encroachment onto vital structures thus preventing any severe iatrogenic morbidity. In case of an active orbital fungal invasion, exenteration might be lifesaving. Many studies have come to a conclusion that exenteration should be considered for an actively inflamed orbit with an immobile blind eye [33]. Even after the intracranial spread has occurred, orbital exenteration conducted with the motive of decreasing the fungal load is still helpful.

For medical management of Mucormycosis, Amphotericin B (AmB) remains the mainstay of treatment. Though, Amphotericin B deoxycholate is the crux of treatment its usage is associated with renal toxicity therefore, a careful monitoring of blood urea nitrogen, serum electrolytes, serum creatinine along with creatinine clearance should be done. An infectious disease specialist or physician should be kept in loop to avoid any untoward reaction.

### Outcomes

Appropriate diagnosis and timely intervention is quintessential for better outcomes.

Prognosis of invasive fungal rhino sinusitis patients remains poor despite aggressive surgery and intravenous anti-fungal therapy, with documented mortality rates of 33.3–80%, which may go up to 100% in disseminated infections.

## Recommendations

- In diabetic patients or patients being treated for COVID illness, the blood pH should be maintained above 7.35 in order to avoid an acidotic state which promotes growth of mucor.
- We should refrain ourselves from labelling every COVID-positive case presenting with symptoms like diminution of vision/headache as a case of mucor, it maybe a commensal fungus also.
- We recommend doing CT scan and MRI for every suspected case of sinonasal infection in a COVID-positive patient. MRI will help us in ruling out intraorbital and intracranial extension/cerebral venous thrombosis. CT scan will help us in mapping the disease and planning our surgery.
- Based on our experience, we, in consultation with ophthalmologist, should plan orbital exenteration at optimal time. We advocate opportune surgical intervention for intraorbital extension and a more aggressive approach so as to remove the dead tissue completely, hence improving overall chance of survival.
- Role of pathologist and microbiologist is of paramount importance in timely diagnosis of such cases. Remember all complicated rhinosinusitis in COVID-positive patients may not be mucormycosis. Any specific treatment for rhinocerebro-orbital mucormycosis should be started after a positive fungal stain report.
- The role of infectious disease specialist is very important. Appropriate anti-fungal therapy should be started after consulting infectious disease specialist. Correct dose and duration of drugs such as Amphotericin B is very crucial in treating mucor patients and also to prevent or minimize drug toxicity.
- The role of intensivist in providing oxygen support to COVID-positive patients can prove to be a major factor in reducing incidence of sinonasal infections in these patients. The mode of delivery of oxygen, rate of delivery, level of humidification needed and tapering of oxygen requirement in such patients based on their recovery will prove very useful in maintaining a correct pH, thus preventing the growth of opportunistic agents.
- The management of complicated rhinosinusitis in COVID era involves a multidisciplinary team effort.

## Conclusion

Complicated sinusitis was encountered in COVID-positive patients either when they were being actively treated for COVID-19 or as part of post-COVID sequalae. Though rhino-orbito-cerebral mucormycosis constituted the major disease burden in such patients but the possibility of rhino sinusitis with or without complications must also be kept in mind while evaluating such patients. We must remember every complicated rhinosinusitis in COVID-positive patient may not be mucor.

## Data Availability

The datasets used and analyzed during this study are included in the published article and also are available from the corresponding author on reasonable request.

## Abbreviations

SARS-CoV-2: Severe acute respiratory syndrome coronavirus 2
CT: Computed tomography
MRI: Magnetic resonance imaging
RT-PCR: Reverse transcriptase polymerase chain reaction
ENT: Ear, nose, and throat
FESS: Functional endoscopic sinus surgery
IFRS: Invasive fungal rhinosinusitis
EESS: Extended endoscopic sinus surgery
HPE: Histopathological examination
ITF: Infratemporal fossa
GC: Glucocorticoids
HSCT: Hematopoietic stem cell transplant
ACE2: Angiotensin converting enzyme 2
DNE: Diagnostic nasal endoscopy
AmB: Amphotericin B
PNS: Paranasal sinuses
